# Interactions of *Mycoplasma hyopneumoniae* and/or *Mycoplasma hyorhinis* with *Streptococcus suis* Serotype 2 Using In Vitro Co-Infection Models with Swine Cells

**DOI:** 10.3390/pathogens12070866

**Published:** 2023-06-22

**Authors:** Héloïse Pageaut, Sonia Lacouture, Mélanie Lehoux, Corinne Marois-Créhan, Mariela Segura, Marcelo Gottschalk

**Affiliations:** 1 Swine and Poultry Infectious Diseases Research Center (CRIPA) and Groupe de Recherche sur les Maladies Infectieuses en Production Animale, Faculty of Veterinary Medicine, University of Montreal, 3200 Sicotte St., Saint-Hyacinthe, QC J2S 2M2, Canada; heloise.pageaut@umontreal.ca (H.P.); sonia.lacouture@umontreal.ca (S.L.); melanie.lehoux@umontreal.ca (M.L.); mariela.segura@umontreal.ca (M.S.); 2 Ploufragan-Plouzané-Niort Laboratory, Mycoplasmology Bacteriology and Antimicrobial Resistance Unit, French Agency for Food, Environmental and Occupational Health & Safety (ANSES), 22 440 Ploufragan, France; corinne.marois@anses.fr

**Keywords:** *Streptococcus suis*, *Mycoplasma hyopneumoniae*, *Mycoplasma hyorhinis*, co-infections, in vitro, toxicity, inflammation, alveolar macrophages, dendritic cells, tracheal epithelial cells

## Abstract

Bacterial and/or viral co-infections are very common in swine production and cause severe economic losses. *Mycoplasma hyopneumoniae*, *Mycoplasma hyorhinis* and *Streptococcus suis* are pathogenic bacteria that may be found simultaneously in the respiratory tracts of pigs. In the present study, the interactions of *S. suis* with epithelial and phagocytic cells in the presence or absence of a pre-infection with *M. hyopneumoniae* and/or *M. hyorhinis* were studied. Results showed relatively limited interactions between these pathogens. A previous infection with one or both mycoplasmas did not influence the adhesion or invasion properties of *S. suis* in epithelial cells or its resistance to phagocytosis (including intracellular survival) by macrophages and dendritic cells. The most important effect observed during the co-infection was a clear increment in toxicity for the cells. An increase in the relative expression of the pro-inflammatory cytokines IL-6 and CXCL8 was also observed; however, this was the consequence of an additive effect due to the presence of different pathogens rather than a synergic effect. It may be hypothesized that if one or both mycoplasmas are present along with *S. suis* in the lower respiratory tract at the same time, then increased damage to epithelial cells and phagocytes, as well as an increased release of pro-inflammatory cytokines, may eventually enhance the invasive properties of *S. suis*. However, more studies should be carried out to confirm this hypothesis.

## 1. Introduction

Worldwide, *Streptococcus suis* infections spread and are one of the most important causes of post-weaning mortality in pigs [1]. *S. suis*-associated diseases do not only result in severe economic losses but also raise animal welfare concerns. In addition, they are difficult to control [2]. Even if the carriage rate of *S. suis* in clinically healthy animals is high, the incidence of disease varies from period to period and is generally less than 5% [3]. However, this is usually the case when antimicrobials are used (if allowed) as prophylactic/metaphylactic measures; otherwise, the mortality may rise up to 20%. One of the main problems is that antimicrobials that have efficacy against *S. suis* are those that the industry is trying to reduce given their importance in both human and veterinary medicine.

Although highly virulent strains of *S. suis* have been reported, mainly belonging to serotype 2 [4], a full definition of what constitutes an “*S. suis*-associated disease” is complex. Indeed, the development of clinical signs may also depend on external factors, such as the environment and management practices [2,5]. Indeed, *S. suis* is a normal inhabitant of the upper respiratory tracts of pigs, and the presence of virulent strains alone does not guarantee the development of clinical signs, while the latter may appear in the absence of highly virulent strains [5]. The conditions permitting certain serotypes/strains of *S. suis* to cross the mucosal barriers, disseminate in the bloodstream and cause systemic infections are not fully understood [1]. A plethora of potential virulence factors have been described, although, for many, there is still debate on their actual roles in the pathogenesis of *S. suis* infection [4,5]. It is believed that, under some circumstances, *S. suis* takes advantage of concomitant or previous infections caused by other pathogens. However, few studies have clearly demonstrated the role of co-infections in the development of *S. suis*-associated diseases [5]. Among them, only co-infections with either the porcine reproductive and respiratory syndrome virus (PRRSv) or the swine influenza virus (SIV) have been shown to influence the presence of *S. suis*-associated diseases [5,6]. Other studies have addressed the possible effect of the co-infection of *S. suis* with porcine circovirus type 2 (PCV2), although this has not been definitively confirmed [5].

Although many studies have addressed the role of *S. suis* in respiratory diseases, the route of entry of a pathogen should be differentiated from the pathology it induces. It is widely accepted that the main route of infection for systemic *S. suis* disease is the respiratory route, and airborne transmission of the infection has clearly been demonstrated [1]. Infectious agents involved in the porcine respiratory disease complex (PRDC) are classified into primary, secondary or opportunistic pathogens. As a respiratory pathogen, *S. suis* is usually considered a secondary/opportunistic agent [5,7]. As shown with PRRSv and SIV, interactions with respiratory viruses may influence the invasive properties of *S. suis* and increase the severity of systemic-*S. suis*-associated diseases [5].

*Mycoplasma hyopneumoniae* is the etiological agent of enzootic pneumonia in growing and late-growing pigs. The disease is characterized by increased morbidity and decreased daily weight gain in pigs, resulting in important economic losses [8,9]. *M. hyopneumoniae* is also one of the initiating agents of the PRDC, causing cough, fever, loss of weight and appetite, lethargy and sometimes death, especially in cases of superinfections with other pathogens [9,10]. *Mycoplasma hyorhinis* is responsible for inflammation of the serosa in post-weaned piglets leading to pericarditis, pleurisy, arthritis and peritonitis [11,12]. Disease induced by *M. hyorhinis* infection can lead to high morbidity, lameness and weight loss in the herd, also resulting in economic losses. *M. hyorhinis* is often found in lungs in the presence of *M. hyopneumoniae*, but its role as a primary agent of pneumonia remains controversial [13]. Cases of co-infection of *M. hyopneumoniae* with PRRSv, PCV2, SIV or *Actinobacillus pleuropneumoniae*, as well as co-infections with *M. hyorhinis* and PRRSv, PCV2 or *M. hyopneumoniae*, have been described and shown to often lead to increased clinical signs and inflammatory response [7]. The simultaneous isolation from the pericardial tissue of *S. suis* and both of these mycoplasmas has been described [14]. In addition, there is one report indicating a possible correlation between the presence of *S. suis* and *M. hyopneumoniae* in the lungs [15], while a second study describes the simultaneous detection of *M. hyorhinis* and *S. suis* from cases of polyserositis [16]. However, the interactions between *M. hyopneumoniae* and/or *M. hyorhinis* with *S. suis* have never been studied.

All three pathogenic bacteria are present in the respiratory tract of pigs and may induce an inflammatory response in the host. It is therefore interesting to study their possible interaction and mechanisms involved in co-infections. In the current study, we aimed to evaluate the effect of a pre-infection with *M. hyopneumoniae* and/or *M. hyorhinis* on the interactions of *S. suis* serotype 2 with swine epithelial and phagocytic cells.

## 2. Materials and Methods

### 2.1. Bacterial Strains and Growth Conditions

Two strains of *S. suis* serotype 2 were used in this study (Table 1): the reference virulent, encapsulated P1/7 strain (sequence type 1), isolated in Europe, and, as a control, its derived isogenic non-encapsulated mutant *∆cps2F* [17]. Bacteria were grown overnight on Columbia agar supplemented with 5% sheep blood (Oxoid, Burlington, ON, Canada) at 37 °C under 5% CO_2_. An amount of 5 mL of Todd Hewitt Broth (THB) (Becton Dickinson, Mississauga, ON, Canada) was inoculated with a few colonies of *S. suis* and incubated for 16 h at 37 °C under 5% CO_2_. For the final culture, 10 mL of fresh medium was inoculated with 300 µL of the overnight culture and incubated at 37 °C under 5% CO_2_ until the exponential growth phase corresponding to an optical density of 0.6 (OD_600_) was reached. The cultures were then centrifuged, and the bacterial pellets were washed twice with endotoxin (lipopolysaccharide)-free phosphate-buffered saline (LPS-free PBS).

Two strains of porcine mycoplasmas were also used in this study (Table 1): *M. hyopneumoniae* strain 682 and *M. hyorhinis* strain 380, both recovered from pig lungs with macroscopic pneumonia-like lesions [18]. Both strains were grown in liquid Friis medium, as described [19], at 37 °C, until the onset of the stationary phase of growth that is observed in the change from a red to orange color of the medium using the phenol red pH indicator. *Mycoplasma* cultures were centrifuged at 12,000× *g* for 15 min at 4 °C. The pellets were washed twice with LPS-free PBS, and then resuspended with the different cell culture media. The final concentration of both mycoplasmas was determined by quantitative Polymerase Chain Reaction (qPCR) and confirmed by titration in Friis medium. The qPCR was performed as previously described [20], with a few modifications. Briefly, the culture was centrifuged, and the pellet resuspended in the lysis solution described by Kellog and Kwok [21]. The cell lysates were prepared via incubation for 1 h at 60 °C, followed by a 15 min incubation at 95 °C. The primers used are listed in Table 2. Each PCR reaction was performed using 5 µL of DNA template, 20 µL of reaction mixture containing 1 × iQ Supermix (Biorad Laboratories, Hercules, CA, USA), 500 nmol L^−1^ of primers and 300 nmol L^−1^ of hydrolysis probes (TaqMan^®^ Technology, Sigma-Aldrich^®^, Saint-Quentin Fallavier, France). The amplification was performed with the CFX96 thermal cycler (Biorad), and the cDNA amplification program was performed as follows: a 3 min enzyme activation step at 95 °C, followed by 40 cycles of denaturation at 95 °C for 15 s and a 1 min annealing/extension step at 60 °C.

**Table 1:** List of strains used in this study.

Strains	Characteristics	References
***S. suis* serotype 2 ** P1/7 *∆cps2F*	Virulent serotype 2 strain isolated from a case of pig meningitis in the United Kingdom Non-encapsulated isogenic mutant derived from P1/7; in-frame deletion of *cps2F* gene	[22,23]
***Mycoplasma*** *M. hyopneumoniae* 682 *M. hyorhinis* 380	Strains isolated in 2016 from the same herd showing respiratory disorders	[18]

### 2.2. Source of Primary Cells

Piglets between 5 and 8 weeks of age used for the isolation of primary cells in this study were negative for various commonly found diseases, such as PRRSv and *M. hyopneumoniae*. No endemic clinical infections caused by *S. suis* serotype 2 or *M. hyorhinis* were present in the herd of origin. All animal experiments were conducted in accordance with the ethical guidelines and policies of the Canadian Council of Animal Care and specifically approved by the Animal Welfare Committee of the University of Montreal (certificate number RECH-1570).

### 2.3. Cell Culture: Newborn Pig Tracheal Cell Line (NPTr), Primary Pulmonary Alveolar Macrophages (PAMs) and Bone-Marrow-Derived Porcine Dendritic Cells (BM-DCs)

NPTr cells were obtained from Dr. M. Jacques, University of Montreal. They were grown as described [24,25,26]. Briefly, cells were grown at 37 °C under 5% CO_2_ in Dulbecco’s Minimum Essential Medium (DMEM) (Gibco, Burlington, ON, Canada), supplemented with 10% heat-inactivated fetal bovine serum (*v/v*) (FBS) (Gibco), penicillin–streptomycin at 100 U/mL and gentamicin at 0.04 mg/mL (Gibco). For the experiments, cells were treated with 0.1% Trypsin in 0.03% ethylenediaminetetracetic acid (EDTA) (Gibco), suspended in fresh culture medium with antibiotics and incubated in a 24-well culture plate (Falcon, Mississauga, ON, Canada) for 24 h at 37 °C under 5% CO_2_. After 24 h, the cells were washed once with LPS-free PBS, and the culture medium was replaced with fresh antibiotic-free culture medium. The cells were again incubated for 24 h until confluence was achieved with a final concentration of 1 × 10^5^ cells/mL.

To collect alveolar macrophages [24], three successive bronchoalveolar washes were performed with sterile LPS-free PBS in the lungs of 5–8-week-old piglets without the use of antibiotics. Cells were washed twice and then frozen in liquid nitrogen with DMEM medium supplemented with 40% FBS and 20% dimethyl sulfoxide (DMSO) (Sigma-Aldrich, St. Louis, MO, USA) to a final concentration of 2 × 10^7^ cells/mL. To confirm the sterility of the cell harvest, PAMs were incubated for 48 h under 5% CO_2_ in a Primaria 24-well plate (Falcon BD, Bedford, MA, USA). For assays, PAMs were thawed, centrifuged at 250× *g* for 10 min at 4 °C and suspended in antibiotic-free PAM medium containing DMEM, 10% FBS, 1% MEM Non-Essential Amino Acid Solution and 1% HEPES (Gibco) at a concentration of 5 × 10^5^ cells/mL in a Primaria 24-well plate and incubated at 37 °C under 5% CO_2_.

The extraction and culture of cells from the bone marrow of pig femurs was performed as described [18,27]. Briefly, after removal of muscles, femurs were sliced into pieces and shaken in 1 L of LPS-free PBS for 2 h at room temperature. The suspension containing the bone marrow stem cells was recovered by filtration using sterile gas, and the cells were then centrifuged at 250× *g* for 10 min at 4 °C. Red blood cell lysis was performed with a kit (eBioScience, San Diego, CA, USA), then cells were washed, filtered on a 40 µm cell strainer (Falcon), suspended at approximately 2 × 10^7^ cells/mL in cryopreservation solution containing 95% FBS and 5% DMSO and stored in liquid nitrogen. For the experiments, bone-marrow-derived stem cells were thawed and centrifuged at 250× *g* for 10 min at 4 °C. Cells were suspended in a complete medium with antibiotics containing RPMI 1640 (Gibco), 10% FBS (*v/v*), 10 mM HEPES, 2 mM L-glutamine (Gibco), 0.001 mg/mL gentamicin and 100 U/mL penicillin–streptomycin at a concentration of 1 × 10^6^ cells/mL in a 6-well plate (Falcon). Porcine Granulocyte-Macrophage Colony Stimulating Factor (GM-CSF), produced in our laboratory from the CHO-K1/pGM-CSF cell line as previously described [27], was added at a 1:50 dilution to each well, and the cells were incubated at 37 °C under 5% CO_2_. On days 3 and 6, the culture medium was replaced with complete RPMI medium fresh with antibiotics to remove non-adherent cells. On day 8, immature BM-DCs were harvested via up and down pipetting for future experiments. The phenotype of BM-DCs was confirmed by FACS as CMH-I^+^, CMH-II^+^, SWC3^+^, CD1^+^, CD16^+^, CD14^+^, CD11R1^−^ and CD4^a low/−^, as previously described [28]. This culture does not exclude the presence of other cells, such as macrophages, but the culture is predominantly enriched in immature BM-DCs. Immature BM-DCs were adjusted to a concentration of 2 × 10^6^ cells/well in a total volume of 500 µL in a 24-well plate (Falcon).

When needed, fresh or inactivated swine plasma was used. Blood from a 5-week-old piglet was collected in heparinized tubes to avoid clotting. The blood was centrifuged, and the plasma was recovered and stored at −80 °C to maintain the integrity of the complement system. The plasma had a negative serological status for antibodies against *S. suis* as tested by ELISA [29]. Fresh plasma and heat-inactivated plasma (30 min at 56 °C) were used for the phagocytosis and intracellular survival experiments described below.

### 2.4. Pre-Infection and Stimulation of NPTr Cells, PAMs and BM-DCs with M. hyopneumoniae and/or M. hyorhinis

To determine whether porcine mycoplasmas promote *S. suis* interactions with respiratory epithelial cells, NPTr cells were first pre-infected with *M. hyopneumoniae* or *M. hyorhinis* or both (or mock-pre-infected) under non-cytotoxic conditions as follows. NPTr cells were washed twice with LPS-free PBS, and 1 mL of *M. hyopneumoniae* suspension of approximately 1 × 10^8^ color-changing units per milliliter (CCU/mL) was added to the wells. In parallel, 500 µL of *M. hyorhinis* suspension and 500 µL of medium without antibiotics for a final concentration in the wells of approximately 5 × 10^6^ CCU/mL was also added to cells. For the dual infection (pre-infection with *M. hyopneumoniae* and *M. hyorhinis*), the mycoplasma suspensions were concentrated twice and 500 µL of *M. hyopneumoniae* was added to the cells together with 250 µL of *M. hyorhinis* and 250 µL of fresh antibiotic-free culture medium. The mock-pre-infected control cells were given fresh culture medium without antibiotics. The cells were centrifuged for 10 min and then incubated for 24 h at 37 °C under 5% CO_2_. The final concentration of *M. hyopneumoniae* and *M. hyorhinis* was determined by qPCR and confirmed by titration in Friis medium, as described above.

For pre-infection of PAMs, the culture medium was removed, and the cells were pre-infected with 1 mL of either 1 × 10^7^ CCU/mL of *M. hyopneumoniae* or 1 × 10^6^ CCU/mL of *M. hyorhinis* suspensions for single infections. For the double infection, the mycoplasma suspensions were concentrated twice (respectively) and 500 µL of each strain was added to the cells. The mock-pre-infected cells were given fresh culture medium without antibiotics. As for NPTr, PAMs were centrifuged and then incubated for 24 h at 37 °C under 5% CO_2_.

Finally, the culture medium of the BM-DCs was removed, and the cells were pre-infected with 500 µL of either *M. hyopneumoniae* or *M. hyorhinis* suspensions in a medium without antibiotics, for final concentrations of 5 × 10^7^ CCU/mL and 5 × 10^5^ CCU/mL for mono-infection, respectively. For the double infection, the mycoplasma suspensions were concentrated twice, and 250 µL of each strain was added to the cells followed by 500 µL of fresh culture medium without antibiotics. The mock-pre-infected cells were given fresh culture medium without antibiotics. BM-DCs were centrifuged as described above and then incubated for 24 h at 37 °C under 5% CO_2_.

The final concentrations of each mycoplasma were chosen based on pre-studies to obtain the highest concentration with the lowest percentage of cytotoxicity (from 0 to 15%, depending on the cell type) after 24 h of incubation using the lactate dehydrogenase test, as described below (results not shown). At the end of 24 h incubation time, the concentrations of both mycoplasmas either remained stable or increased, indicating that live mycoplasmas were present at the time of *S. suis* infection (Supplemental Table S1).

### 2.5. Adhesion to and Invasion of NPTr by S. suis

After 24 h of pre-infection with the mycoplasmas (or mock-pre-infected cells), NPTr cell culture medium was removed from the wells. The cells were then infected with either the *S. suis* wild-type P1/7 strain or its unencapsulated *∆cps2F* mutant (positive control) at a multiplicity of infection (MOI) of 5 (5 × 10^5^ colony-forming units (CFU)/mL), fresh medium without antibiotics was added to the cells and the plates were incubated for 2 h at 37 °C under 5% CO_2_. The MOI used was based on preliminary studies showing the highest non-toxic concentration of *S. suis* after a pre-infection with both mycoplasmas. An *S. suis* adhesion assay was performed as previously described [24,25]. Briefly, after the incubation time, cells were washed 5 times with LPS-free PBS and scraped with 1 mL of ice-cold sterile deionized water to recover adhering as well as intracellular bacteria. The bacterial suspensions were plated on Todd Hewitt agar (THA) (Gibco) and incubated for 24 h at 37 °C under 5% CO_2_. The adhesion rate of *S. suis* was expressed as CFU/mL. For the *S. suis* invasion assay, cells were washed twice with LPS-free PBS. Then, 1 mL of fresh culture medium with antibiotics (penicillin G (5 µg/mL) and gentamicin (100 µg/mL)) was added to each well and incubated for 1 h at 37 °C under 5% CO_2_. Wells were then washed 3 times with LPS-free PBS, and the last wash was spread onto THA to confirm antibiotic activity. The cells were then scraped to count the intracellular bacteria only, and the number of CFU/mL of *S. suis* was determined in the same way as described above. The invasion rate of *S. suis* was also expressed as CFU/mL.

### 2.6. Phagocytosis and Intracellular Survival Assay of S. suis by PAMs and BM-DCs

*S. suis* phagocytosis tests were carried out as described previously [17,24,28], with some modifications. *S. suis* P1/7 strain and its *∆cps2F* (positive control) were pre-opsonized with 20% of either fresh or heat-inactivated swine plasma (30 min at 37 °C) with shaking [28]. After 24 h of pre-infection with mycoplasmas, the culture medium of the PAMs and BM-DCs was removed, and cells were infected with a final concentration of pre-opsonized 1 × 10^7^ CFU/mL of *S. suis* P1/7 strain or its mutant *∆cps2F* (MOIs of 20 and 10 for PAMs and BM-DCs, respectively). The MOI used was based on preliminary studies showing the highest non-toxic concentration of *S. suis* after a pre-infection with both mycoplasmas. The infected 24-well cell culture plates were centrifuged and incubated for 30 and 60 min at 37 °C under 5% CO_2_ for PAMs and BM-DCs, respectively (optimal time for *S. suis* phagocytosis determined by previous phagocytosis assays). Cells were then washed twice with LPS-free PBS and culture medium with antibiotics (gentamicin (100 µg/mL) and penicillin G (5 µg/mL)) was added to the cells. The cells were incubated for 2 h at 37 °C under 5% CO_2_. After antibiotic treatment, cells were washed three times with LPS-free PBS, and the last wash was plated onto THA to confirm the effectiveness of the antibiotic treatment. The cells were then scraped and the CFU count of the bacteria was determined in the same way as described above. The phagocytosis rate of *S. suis* was expressed as CFU/mL.

To study intracellular survival, bacteria were left for 30 or 60 min to be internalized by the cells (depending on the cell type). As described above, a penicillin G and gentamicin solution was added, but this time cells were cultured for an additional 1 h, 3 h, 5 h and 7 h in the presence of antibiotics. Cells were then processed as described above and the surviving intracellular bacteria counted.

### 2.7. Cytotoxicity Assay

The cytotoxic effects on cells were assessed by measuring the release of lactate dehydrogenase (LDH) enzyme, as previously described [18]. Briefly, mycoplasma pre-infected (or not) cells were then infected with *S. suis* P1/7 strain at an MOI of 10 (NPTr) or 1 (PAMs and BM-DCs). Because the epithelial cells are more resistant than the primary phagocytic cells, a higher MOI was used. Mock-infected cells were also included. The cells were centrifuged and incubated for various time points at 37 °C under 5% CO_2_. Recovered supernatants were used for LDH detection using the CytoTox 96 Non-Radioactive Cytotoxicity Assay kit (Promega, Madison, WI, USA), according to the manufacturer’s instructions.

### 2.8. Induction of Pro-Inflammatory Cytokines

After pre-infection with one or both mycoplasmas, porcine cells were washed and infected with the *S. suis* P1/7 strain at an MOI of 10 (1 × 10^6^ CFU/mL) for NPTr cells or 1 (5 × 10^5^ CFU/mL) for PAMs and BM-DCs (1 × 10^6^ CFU/mL). The MOI used was based on preliminary studies showing the highest non-toxic concentration of *S. suis* after pre-infection with both mycoplasmas. Medium from mock-pre-infected cells was also removed and fresh medium without antibiotics was added to the cells. Plates were centrifuged and incubated for 12 h (NPTr) or 6 h (PAMs and BM-DCs) at 37 °C under 5% CO_2_. After each incubation time, cells were washed once with LPS-free PBS, and RNA from the cells was extracted using a silica column, according to the manufacturer’s instructions (Aurum total RNA mini kit, Biorad, Mississauga, ON, Canada). Briefly, cells were lysed with a solution containing β-mercaptoethanol and 70% ethanol. The cell lysate was transferred to a silica column and washed with low- and high-stringency wash solutions from the kit. DNase solution was added to the cell lysate to retain only the RNA. RNA was eluted into 40 µL of elution solution, quantified using the NanoDrop 1000 (Fisher, Ottawa, ON, Canada), diluted in RNase-free water to 100 ng/µL and stored at −80 °C. Complementary DNA (cDNA) was synthesized from 200 ng of RNA with the M-MLV reverse transcriptase kit (Invitrogen, Carlsbad, CA, USA) using Oligo (dT)_12-18_ Primer 25 µg (0.5 µg/µL) and dNTP Mix, 10 mM each (Thermoscientific, Vilnius, Lithuania), according to the manufacturer’s instructions. 

The primers used for the q-PCR analysis are listed in Table 3. The qPCR was performed as described, with some modifications [24]. Briefly, the CFX96 thermal cycler (Biorad, Canada) showed that the primers had an efficiency between 90 and 110%. The cDNA was amplified using the PowerTrack SYBR Green Master Mix Kit (Thermofisher, Vilnious, Lithuania). The cDNA amplification program was performed as follows: a 2 min enzyme activation step at 95 °C, followed by 40 cycles of 15 s denaturation at 95 °C and a 1 min annealing/extension step at 58 °C. The two genes *β2M* and *PPiA* were used as normalization genes to compensate for potential differences in the cDNA amounts between the different samples. Differences in gene expression were calculated using the normalized gene expression calculation method (∆∆Cq) of CFX Maestro software (v.2.1: Biorad, USA). Uninfected cells were used as negative control and calibrator for the analysis. Results represent at least four independent experiments.

### 2.9. Statistical Analysis

SigmaPlot software (v.11.0) was used for data analysis. Parametric (unpaired *t* test) or non-parametric (Mann–Whitney rank sum test) tests, where appropriate, were performed to evaluate statistical differences between conditions. Each in vitro test was repeated in four independent experiments, with three independent technical replicates in each experiment. *p* < 0.05 was considered the threshold for statistical significance.

## 3. Results

### 3.1. Pre-Infection of NPTr Epithelial Cells with M. hyopneumoniae and/or M. hyorhinis Does Not Promote Adhesion and Invasion of S. suis

*M. hyopneumoniae*, *M. hyorhinis* and *S. suis* colonize the upper respiratory tract of pigs. All three pathogenic bacteria have the ability to adhere to and potentially invade the epithelial cells of the trachea of pigs [1,30,31]. In order to evaluate the effect of mycoplasma pre-infection on the early stages of the pathogenesis of the *S. suis* infection, the rate of adhesion and invasion of *S. suis* on tracheal epithelial cells was studied under non-cytotoxic conditions, as established in preliminary testing (data presented below).

**Figure 1:**
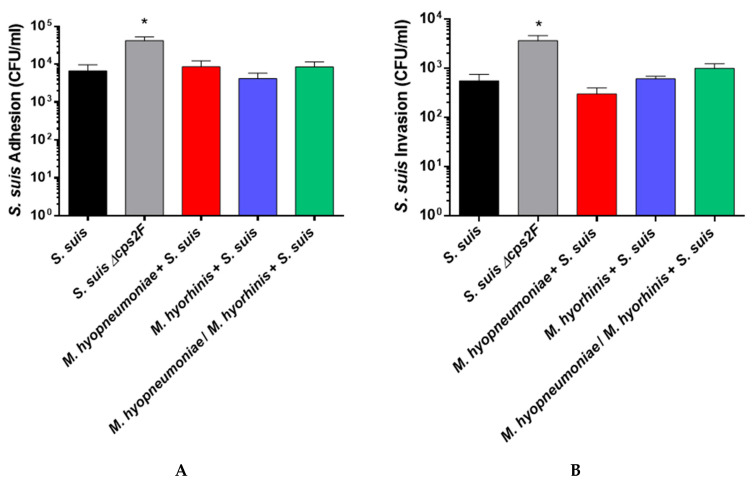
Adhesion and invasion of NPTrs by *S. suis* serotype 2 P1/7 and its *∆cps2F* non-encapsulated mutant. Adhesion (**A**) and invasion (**B**) of the *S. suis* wild-type and mutant strains to NPTr porcine tracheal epithelial cells after 2 h of incubation at an MOI of 5. Data represent the mean ± SEM (*n* = 4, with 3 technical replicates each). * *p* < 0.05 indicates a significant difference between the wild-type and its ∆cps2F mutant strain using a parametric unpaired *t* test.

As shown in Figure 1A, and as expected, the adhesion rate of the ∆cps2F mutant (used as a positive control) was significantly higher (*p* < 0.05) than that of the wild-type strain and similar to those described previously [24,32]. A pre-infection of NPTr cells with *M. hyopneumoniae* and/or *M. hyorhinis* did not significantly modify the adhesion rates of the *S. suis* P1/7 strain when compared to mock-pre-infected cells (Figure 1A). Similarly, the invasion capacity of the *S. suis* P1/7 strain was not affected by pre-infection with either or both mycoplasmas (Figure 1B). As observed in previous studies, a significant difference in the invasion rate between the wild-type P1/7 strain and its ∆cps2F mutant was also found [32]. In conclusion, pre-infection with either or both mycoplasmas does not influence the adhesion/invasion capacities of *S. suis* under the conditions tested.

### 3.2. Pre-Infection of PAMs and BM-DCs with M. hyopneumoniae and/or M. hyorhinis Does Not Affect Phagocytosis of S. suis

**Figure 2:**
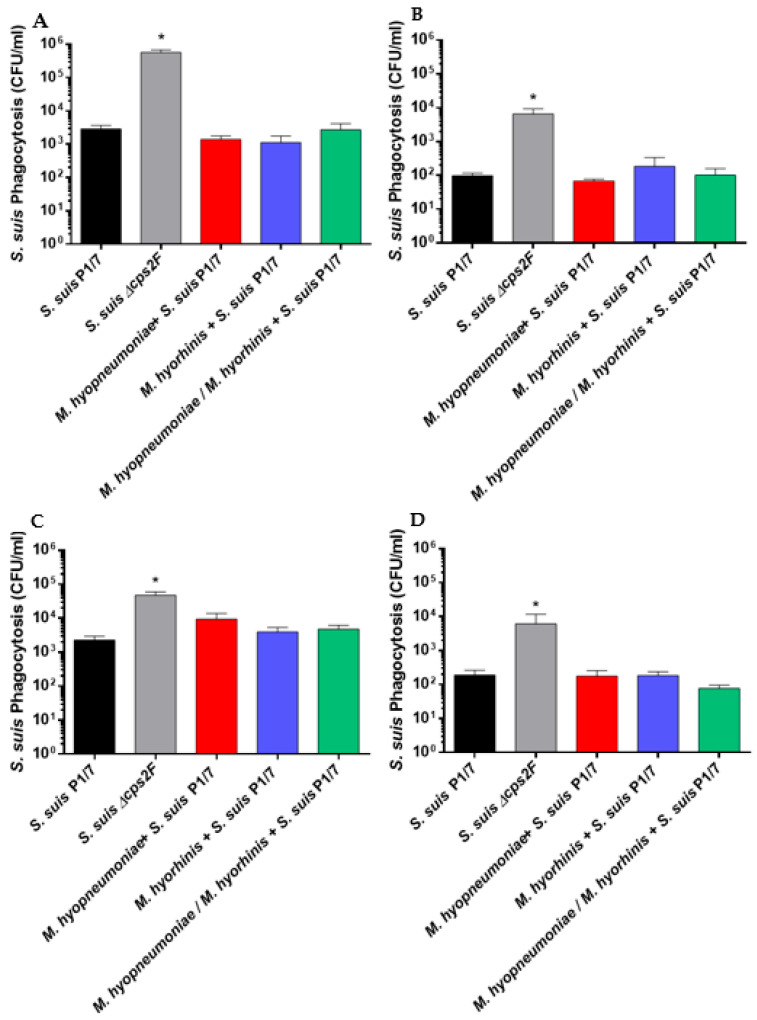
Phagocytosis of *S. suis* serotype 2 strain P1/7 and its *∆cps2F* non-encapsulated mutant by PAMs and BM-DCs. After pre-infection (or not) with *M. hyopneumoniae* and/or *M. hyorhinis*, the cells were infected with *S. suis* for 60 min at an MOI of 20 (PAMs: (**A**,**B**)) and for 30 min at an MOI of 10 (BM-DCs: (**C**,**D**)). The phagocytosis rate of *S. suis* in the presence of fresh porcine plasma (**A**,**C**) or heat-inactivated plasma (**B**,**D**) is expressed as colony-forming units (CFU/mL). Data represent the mean ± SEM (*n* = 4, with 3 technical replicates each). The asterisk shows a significant difference between the wild-type strain P1/7 and its *∆cps2F* mutant strain (*p* < 0.05) using non-parametric Mann–Whitney test.

One of the main roles of alveolar macrophages and dendritic cells is to phagocytose and then process ingested microorganisms [33,34]. Therefore, the rate of phagocytosis of *S. suis* was studied in the presence of PAMs and BM-DCs previously pre-infected (or not) with *M. hyopneumoniae* and/or *M. hyorhinis*. The phagocytosis rates of both cell types were significantly higher in the presence of fresh plasma, confirming the complement involvement in the process (*p* < 0.05) (Figure 2A–D) [35]. Moreover, as previously described, the ∆cps2F non-encapsulated mutant strain was significantly more phagocytosed than its wild-type strain (in the presence of either fresh or heat-inactivated plasma) (*p* < 0.05), indicating a normal behavior of the cells used [17,28]. In the presence of fresh plasma, the phagocytosis of the P1/7 strain was higher when using PAMs when compared to BM-DCs (*p* < 0.05) (Figure 2A,C), also similar to what was previously reported [36]. Mycoplasma or mock-pre-infected PAMs phagocytosed *S. suis* at a similar rate for both cell types and under both conditions used (fresh or inactivated plasma) (Figure 2A–D), indicating that pre-infection with either or both mycoplasmas does not influence the phagocytosis activity of these cells towards *S. suis*.

### 3.3. Pre-Infection of PAMs and BM-DCs with M. hyopneumoniae and/or M. hyorhinis Does Not Affect the Intracellular Survival Rate of S. suis

**Figure 3:**
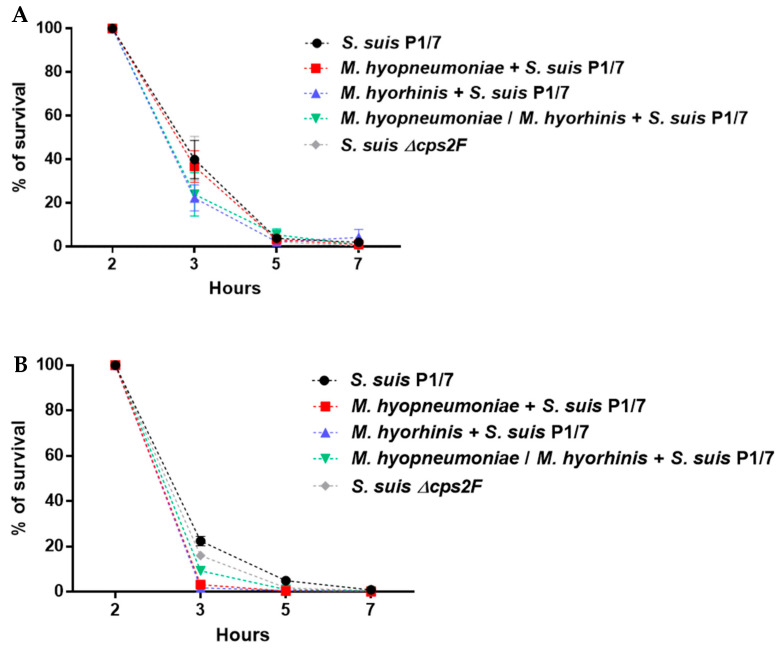
Intracellular survival of *S. suis* serotype 2 strain P1/7 and its *∆cps*2F non-encapsulated mutant within PAMs and BM-DCs. After pre-infection (or not) with *M. hyopneumoniae* and/or *M. hyorhinis*, cells were infected with *S. suis* for 60 min at an MOI of 20 (PAMs: (**A**)) and for 30 min at an MOI of 10 (BM-DCs: (**B**)) in the presence of fresh porcine plasma. Antibiotics were then added for 3 h, 5 h and 7 h. The survival rate of 100% represents the phagocytosis rate (similar to that observed in the previous experiment, Figure 2) after 2 h of antibiotic treatment. The intracellular survival rate of *S. suis* is expressed as colony-forming units (CFU/mL). Data represent the mean ± SEM (*n* = 4, with 3 technical replicates each). No significative differences (*p* > 0.05) were observed using non-parametric Mann–Whitney test.

Resistance to the intracellular killing mechanisms of phagocytes may contribute to the pathogenesis of the infection. Therefore, the intracellular survival rate of *S. suis* in the presence of fresh plasma was assessed. To compare the intracellular survival rate of *S. suis* P1/7 or *∆cps2F*, a value of 100% was assigned to the phagocytosis rate observed after 60 or 30 min of incubation for PAMs and BM-DCs, respectively, followed by 2 h of antibiotic treatment. As previously reported [37], no differences in intracellular survival between the wild-type P1/7 and its unencapsulated mutant were observed in either cell type (Figure 3A,B). A pre-infection of PAMs by any or both of the mycoplasmas tested did not increase the intracellular survival of *S. suis* (Figure 3A). Moreover, after 3 h of antibiotic treatment, the intracellular survival of the P1/7 strain or its ∆cps2F in PAMs decreased significantly from 100% to about 40%. After 5 h of antibiotic treatment, there were few bacteria left (5% intracellular bacteria), and after 7 h of antibiotic treatment, almost no bacteria were found with an intracellular survival rate of less than 1%. Figure 3B shows similar results observed with BM-DCs. After 3 h of incubation, survival was at least 50% lower than that observed for PAMs. Survival rates after 5 h and 7 h of incubation were similar to those observed for PAMs. Pre-infection with mycoplasma did not increase the intracellular survival of *S. suis*.

### 3.4. Pre-Infection of NPTr, PAMs and BM-DCs with M. hyopneumoniae and/or M. hyorhinis Significantly Increases Cytotoxicity

It has been shown that *M. hyopneumoniae* can adhere to the surfaces of epithelial cells, damage the host epithelial barrier and weaken these cells [30,38]. Therefore, we next analyzed the cytotoxic effect of pre-infection with *M. hyopneumoniae* and/or *M. hyorhinis* and subsequent infection with *S. suis* on epithelial cells, PAM cells and BM-DCs.

**Figure 4:**
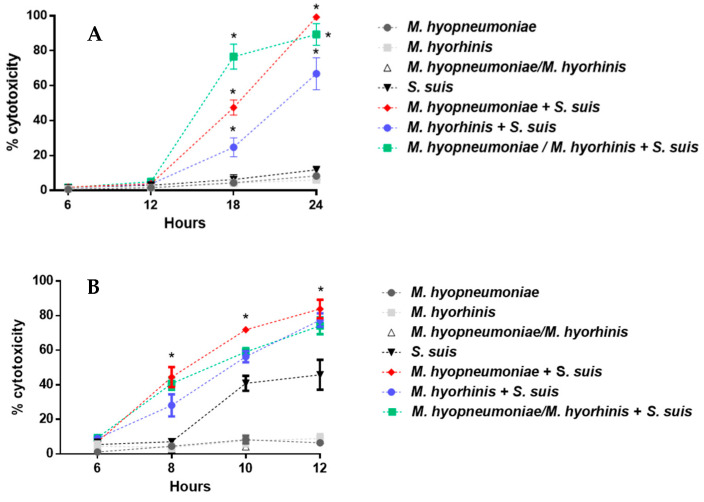
Effect of a pre-infection of mycoplasmas on the cytotoxic effect of *S. suis* on NPTr and PAMs. After pre-infection (or not) with *M. hyopneumoniae* and/or *M. hyorhinis*, cells were infected with *S. suis* P1/7 strain from 6 h to 24 h for NPTr (**A**) at an MOI of 10, and from 6 h to 12 h for PAMs (**B**) at an MOI of 1. Data represent mean ± SEM (*n* = 4, with 3 technical replicates each). The asterisk shows a significant difference between cells pre-infected with mycoplasmas + *S. suis* and cells infected with *S. suis* only or cells pre-infected with mycoplasma only (*p* < 0.05) using non-parametric Mann–Whitney test.

When tested with NPTr cells, at the concentration used, cells infected with one or both mycoplasmas presented a low percentage of cytotoxicity (around 5%) when they were not co-infected with *S. suis* (Figure 4A). Cells infected with only *S. suis* showed a low percentage of cytotoxicity after 6 h, 12 h, 18 h and 24 h of incubation, with means of 1.3%, 2.9%, 6.2% and 11.8% of toxicity, respectively (Figure 4A). In contrast, cells pre-infected with mycoplasmas and then infected with *S. suis* showed significantly increased cytotoxicity (*p* < 0.05) compared to single infections (*S. suis* or mycoplasmas) at 18 h and 24 h of incubation, reaching levels higher than 80% of toxicity. Interestingly, after 24 h of incubation, the percentage of cytotoxicity of the cells pre-infected with *M. hyopneumoniae* or with both mycoplasmas and then co-infected with *S. suis* presented a higher level of cytotoxicity when compared to those co-infected with *M. hyorhinis* and *S. suis* (*p* < 0.05) (Figure 4A).

Similar to epithelial cells, PAMs pre-infected with either or both mycoplasmas presented a low percentage of cytotoxicity (Figure 4B). A low percentage of cytotoxicity (less than 10%) was also found for cells infected with *S. suis,* but, different from epithelial cells, only until 8 h of incubation. However, at later time points, *S. suis*-infected PAMs showed an increased cytotoxicity (approximately 45%) (Figure 4B). Interestingly, there was a rapid and significantly higher increase in cytotoxicity when cells were pre-infected with either of the mycoplasmas (or both) and then infected with *S. suis*. At 10 h of incubation after the infection with *S. suis*, cells pre-infected with *M. hyopneumoniae* presented the highest cytotoxicity level when compared to those infected with *M. hyorhinis* or both. At 12 h of incubation, all mycoplasma pre-infected cells (and co-infected with *S. suis*) presented similar and high cytotoxicity (>75%) (Figure 4B). 

The effect of pre-infection with mycoplasmas on a subsequent infection with *S. suis* of BM-DCs could not be evaluated. In fact, between 6 h and 8 h after infection with *S. suis* (with or without pre-infection with mycoplasmas), a cytotoxicity of higher than 50% was observed. This was mainly due to the high susceptibility of these cells in vitro (Supplemental Figure S1).

### 3.5. Pre-Infection with Mycoplasmas Followed by S. suis Infection of NPTr, PAMs and BM-DCs Differently Modulates the Relative mRNA Expression of Pro-Inflammatory Cytokines

**Figure 5:**
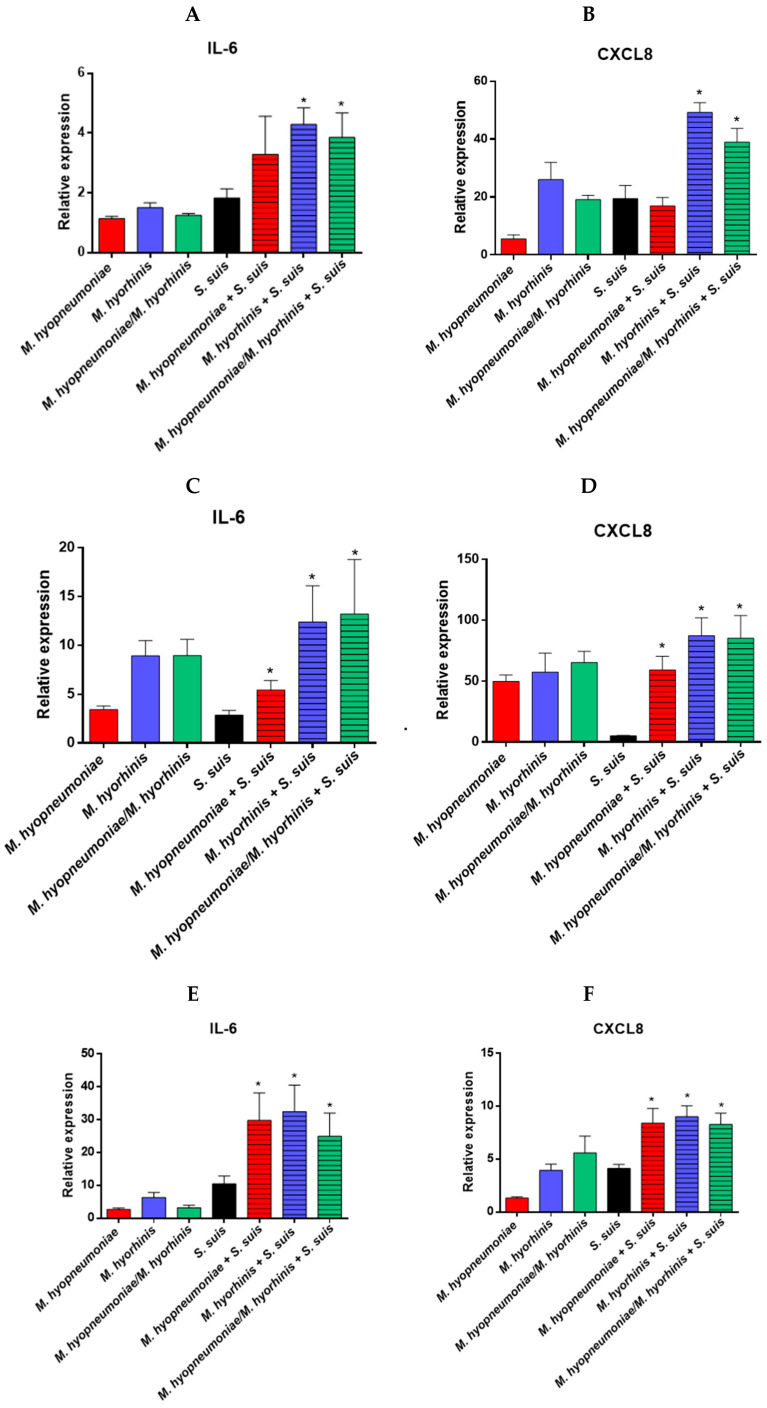
Relative expression of IL-6 and CXCL-8 cytokines expressed by NPTr cells (**A**,**B**), PAMs (**C**,**D**) and BM-DCs (**E**,**F**) by RT-qPCR. After pre-infection of porcine cells with *M. hyopneumoniae* and/or *M. hyorhinis*, cells were infected with *S. suis* P1/7 strain for 12 h at an MOI of 10 (NPTr) and for 6 h at an MOI of 1 (BM-DCs and PAMs). Data represent mean ± SEM (*n* = 4, with 3 technical replicates each). * *p* < 0.05 shows a significant difference between cells infected with *S. suis* only and cells pre-infected with *M. hyopneumoniae* and/or *M. hyorhinis* and then infected with *S. suis* using non-parametric Mann–Whitney test.

In general, epithelial cells induced a similar expression of IL-6 when stimulated either with *S. suis* alone or *M. hyopneumoniae* and/or *M. hyorhinis* alone (Figure 5A). Pre-infection with *M. hyopneumoniae* followed by activation by *S. suis* did not induce a higher expression of this cytokine when compared to cells activated with *S. suis* alone (*p* > 0.05) (Figure 5A). However, a significantly higher mRNA induction of this cytokine was observed when cells were pre-infected with *M. hyorhinis* or both mycoplasmas than activation with *S. suis* alone (Figure 5A). In the case of CXCL8, in general, a higher expression of mRNA was observed when compared to that of IL-6 (Figure 5A,B). However, *S. suis* (as well as *M. hyorhinis* or both mycoplasmas together) induced a significantly higher level of this chemokine than *M. hyopneumoniae* alone (*p* < 0.05). Cells pre-infected with *M. hyorhinis* alone, but not with *M. hyopneumoniae* alone, as well as cells pre-infected with both mycoplasmas, and then infected with *S. suis*, induced higher levels of CXCL8 mRNA than those activated by *S. suis* only (*p* < 0.05) (Figure 5B). However, for both cytokines, the observed activation was likely additive (the sum of mRNA levels induced by each pathogen) rather than synergistic (levels of mRNA of mixed infections significantly higher than the sum of individual levels induced by each pathogen) (Figure 5A). 

As expected, the activation of PAMs showed a clear higher induction of cytokines than epithelial cells (Figure 5C,D). Interestingly, PAMs infected with *M. hyorhinis* (but not *M. hyopneumoniae*) alone, or both mycoplasmas together, induced a higher level of IL-6 than PAMs infected only with *S. suis* (Figure 5C). Cells co-infected with mycoplasmas (each alone or in combination) and *S. suis* induced higher levels of IL-6 than those infected with *S. suis* alone, although this difference was likely additive, and most of the activation appears to have been caused by *M. hyorhinis*. In the case of CXCL8, both mycoplasmas, each alone or in combination, induced similar levels of this chemokine, which were significantly higher than those induced by *S. suis* alone (*p* < 0.05) (Figure 5D). The results showed, in general, that *M. hyopneumoniae* has an additive effect in the inflammatory response in the presence of *S. suis*, whereas the co-infection of *M. hyorhinis* (with either *S. suis* or *M. hyopneumoniae*) induced an additive or synergistic effect on the expression of the inflammation factors. 

Pre-infection of BM-DCs by one or both mycoplasmas induced, in general, similar levels of IL-6 and CXCL8 to those induced by *S. suis* alone (Figure 5E,F). For IL-6, a synergistic effect was observed when cells were pre-activated by either (or both) of the mycoplasmas and then co-infected with *S. suis* (Figure 5E). This effect is less clear for the CXCL8 expression by co-infected cells, which seems to be an additive effect (Figure 5F).

## 4. Discussion

Bacterial and/or viral co-infections are frequent on pig farms. These co-infections may, in some circumstances, increase clinical signs, leading to important economic losses. The PRDC includes a range of “primary” and “secondary” pathogens. The large number of pathogens found in animals makes it difficult to understand the role of each one in the induction of respiratory disease, particularly when pathogens invade through the respiratory route but cause systemic diseases, as is the case of *S. suis* [5]. The interactions between microorganisms and the host immune response are complex and not always well understood [12,39], even though the study of microbial co-infections in pigs is a rapidly expanding area of research [7].

In classical *S. suis* infections in pigs, adhesion to and invasion of the respiratory mucosa are considered the first steps in the development of invasive disease. *S. suis* can also adhere to and invade (although this remains controversial) epithelial cells [40]. For the porcine mycoplasmas included in this study, adhesion to respiratory cells is also the first step in the pathogenesis of the infection. Indeed, *M. hyopneumoniae* can adhere to the surfaces of epithelial cells thanks to adhesins, which leads to ciliostasis, followed by the progressive disappearance of cilia, intense mucus production and epithelial cell death [30,38,41]. This process could facilitate the colonization of “secondary” swine pathogens, such as *A. pleuropneumoniae*, *Pasteurella multocida* and *Glaesserella parasuis*, and lead to the severity of pulmonary lesions [39,41]. Several proteins have been involved in the adhesion of *M. hyopneumoniae* to host cells [30,41,42]. Among them, one membrane protein could be responsible for the adhesion of *M. hyopneumoniae* to tracheal epithelial cells [43]. In addition, it has been reported that *M. hyopneumoniae* increases the attachment of *P. multocida* type A to the respiratory epithelium [44,45]. In contrast, little is known about the pathogenesis of *M. hyorhinis,* but some lipoproteins, as well as glyceraldehyde-3-phophate dehydrogenase, found on the surface of *M. hyorhinis*, have been reported to bind to the surfaces of epithelial cells [31,46]. 

*S. suis* adhesion may be enhanced during co-infection with other pathogens. Indeed, a study by Wang et al. [25], which used the same model described in the current investigation, demonstrated that *S. suis* adhesion and invasion were effectively enhanced by SIV pre-infection. However, in the current study, when we assessed the effect of pre-infection with *M. hyopneumoniae* and/or *M. hyorhinis*, we did not observe any significant influence of either or both mycoplasmas on the *S. suis* adhesion to and invasion of tracheal epithelial cells. These results are similar to those previously observed in a co-infection model of *S. suis* and *G. parasuis* [24]. The lack of effect of the pre-infection with the mycoplasmas cannot be explained by the significant mortality of these microorganisms during the 24 h pre-infection period. Indeed, the number of live mycoplasmas did not decrease (and in some cases, increased) before the cells were infected with *S. suis*. However, because the medium used for the cells was not necessarily a mycoplasma-specific medium, we cannot rule out that *M. hyopneumoniae* or *M. hyorhinis* may have not expressed or adequately displayed some factors needed for adhesion. Indeed, a previous study has shown that some *M. hyopneumoniae* genes can be up- or downregulated depending on environmental conditions, such as the culture medium used for growing the strains, or stress [47]. Although adhesion to tracheal epithelial cells has been reported for *M. hyopneumoniae*, the use of a monolayer has its limitations, and these results should be confirmed using other models. Indeed, it would be interesting to confirm possible interactions between *S. suis* and porcine mycoplasmas using an ex vivo model, such as precision-cut lung slices (PCLSs) [48]. It should be noted, however, that the effect of SIV pre-infection on the *S. suis* adhesion to/invasion of epithelial cells could be observed not only with the experimental method used in this study, but also with the ex vivo PCLS model [6,25].

After crossing the respiratory epithelial barrier, *S. suis* disseminates throughout the body [49]. The lungs are organs that are constantly in contact with pathogens, and phagocytic cells (such as PAMs) play an essential role in the innate immune response against pathogens through bacterial phagocytosis and elimination [50]. Dendritic cells are part of the innate immune system that act as sentinels throughout the body; thus, a large proportion of these cells are also found in the lungs [51]. Resistance to phagocytosis and/or intracellular bacterial elimination may contribute to the pathogenesis of the infection. It has been proposed that *S. suis* is able to destabilize the lipid rafts on the surfaces of macrophages, which contain lactosylceramide, preventing the phagocytosis of encapsulated strains [52]. In this way, *S. suis* reduces phagocytosis and remains extracellular [53]. In the current study, we confirmed that the absence of a capsule increases the phagocytosis of *S. suis* by both PAMs and BM-DCs [24,28]. In addition, we also confirmed that the complement plays an important role in the phagocytosis process [28]. There are very few and contradictory studies on the effect of co-infections on the phagocytosis and intracellular survival of *S. suis*. One study reported the impairment of *S. suis* phagocytosis by PRRSv-infected BM-DCs, whereas another study showed increased phagocytosis by the same cell type [17,54]. There are also very few studies on the effect of mycoplasmas on the phagocytic activity of macrophages. A study showed that *M. hyopneumoniae* can adhere to PAMs, but it is almost not phagocytosed [55]. Another study showed that PAMs pre-infected with *M. hyopneumoniae* presented reduced phagocytosis against *A. pleuropneumoniae* [56]. No data are available in the literature on the effect of *M. hyorhinis* on the phagocytosis activity of PAMs; in addition, it is still unknown whether these cells are able to phagocytose this pathogen. Results of the current study showed that a pre-infection with either *M. hyopneumoniae* or *M. hyorhinis* (or both combined) does not influence the phagocytosis rate of *S. suis*.

After phagocytosis, bacteria end up in an intracellular vesicle called a phagolysosome, where they are degraded by numerous enzymes and free radicals, such as reactive oxygen species [33,34]. A previous study showed the altered expression of more than 2000 genes in the genome of PAMs following infection with *M. hyopneumoniae* [57]. It can be hypothesized that porcine mycoplasmas may modify the expression of some PAM genes and thus favor the intracellular survival of *S. suis*. We therefore investigated whether the pre-infection of PAMs and BM-DCs with *M. hyopneumoniae* and/or *M. hyorhinis* could increase the intracellular survival of *S. suis* after phagocytosis. To achieve an optimal phagocytosis rate of *S. suis*, this experiment was performed in the presence of a complement (fresh pig plasma). Our results clearly showed that a pre-infection with mycoplasmas does not influence the intracellular survival of *S. suis*. Indeed, as reported earlier, *S. suis* only survived a few hours, and the absence or presence of its capsular polysaccharide did not influence the survival rate [28]. 

Toxicity towards host cells is important in *S. suis* pathogenesis. Suilysin, a hemolysin produced mainly by virulent strains from Europe and Asia, such as the P1/7 strain used in the current study [4], is used by *S. suis* to damage and invade host cells [40,58]. Although it is not considered a critical virulence factor [59], the toxic effect of suilysin is important in many steps of the pathogenesis of the infection [60]. Two studies using *Bordetella bronchiseptica*–*S. suis* or SIV–*S. suis* co-infection models have shown that co-infection increases the toxicity caused by *S. suis* [61,62]. In the case of *M. hyopneumoniae*, it has long been known that the pathogen adhesion causes ciliostasis and subsequent cilium loss and epithelial cell death [42]. Very few data are available on the cytotoxic effect of *M. hyorhinis*, other than that reported with porcine dendritic cells [18]. In the current study, conditions were established to work under non-toxic conditions 24 h after the infection of cells with one or both mycoplasmas, as the objective was to study the synergistic effect with *S. suis*. A clear increase in the cytotoxic effect was demonstrated when the NPTr cells and PAMs were pre-infected with mycoplasmas and later infected with *S. suis*. In the case of the NPTr, cells were highly resistant to toxicity induced by *S. suis* alone, but after a pre-infection with either or both mycoplasmas, the toxicity highly increased. PAMs were already more susceptible to the effect of *S. suis* alone; however, a pre-infection with mycoplasmas significantly increased the level of toxicity of these cells. *M. hyopneumoniae* cytotoxicity has been considered a virulence mechanism, as the bacterial infection may induce the death/apoptosis of host cells [42]. In the case of *M. hyorhinis*, similar effects may be hypothesized. A reduced viability of epithelial cells and PAMs during a co-infection may open the door for a subsequent systemic invasion of *S. suis*. However, this hypothesis should be confirmed, as the results obtained in the current study refer mainly to in vitro tests in closed environments. In addition, further studies on the role of apoptosis (clearly demonstrated as being induced by *M. hyopneumoniae* [30,63,64]) on the subsequent infection with *S. suis* remain to be performed.

Inflammation is a hallmark of *S. suis* infections [2]. Septicemia with septic shock induced by *S. suis* results from exacerbated inflammation, and the pig or human host may die within hours of infection. In addition, *S. suis* can induce the production of various pro-inflammatory cytokines by porcine, murine and human cells [40]. *M. hyopneumoniae* is also known to induce the stimulation and secretion of pro-inflammatory cytokines by macrophages in vitro and in vivo [8,65]. *M. hyorhinis* lipoproteins induce the production of pro-inflammatory cytokines by monocytes, splenocytes and BM-DCs [18,66,67]. Furthermore, in cases of bacterial and/or viral co-infections, the production of pro-inflammatory cytokines is increased, which could be detrimental for co-infected animals. Indeed, in vitro co-infection studies of PRRSv or SIV and *S. suis* showed an increased production of pro-inflammatory cytokines [17,25]. In vivo co-infection of PRRSv and *M. hyopneumoniae* in pigs also increased the levels of inflammatory cytokines [68]. Taken separately, these three pathogens induce significant inflammation that can lead to serious consequences for the health of the animal [2,12,42]. The question is whether pre-infection of the NPTr, PAMs and BM-DCs with mycoplasmas and then infection with *S. suis* increases the expression of two important pro-inflammatory cytokines, IL-6 and CXCL8. Results showed, in general, an increased activation of the cytokines tested. However, in most cases, this was the consequence of an additive (rather a synergistic) effect of the pathogens during the co-infection. Results also showed that *M. hyorhinis* seems to have a more powerful capacity to induce cytokine expression than *M. hyopneumoniae*, consistent with previous suggestions [18]. Taken together, the in vitro results indicate that it is difficult to conclude that the pre-infection of pig cells with *M. hyopneumoniae* and/or *M. hyorhinis* and subsequent infection with *S. suis* induces a cytokine storm.

## 5. Conclusions

This study showed relatively limited in vitro interactions between *M. hyopneumoniae* and/or *M. hyorhinis* and *S. suis* serotype 2. A previous infection with one or both mycoplasmas did not influence the *S. suis* adhesion to or invasion of epithelial cells or resistance to phagocytosis (including intracellular survival) by macrophages and dendritic cells. The most important observed effect after pre-infection with the mycoplasmas was a clear increase in toxicity towards cells once co-infected with *S. suis*. An increase in the relative expression of the cytokines studied (usually as the consequence of an additive effect due to the presence of different pathogens) was also observed. It may be hypothesized that if one or both mycoplasmas are present along with *S. suis* in the lower respiratory tract at the same time, then increased damage to epithelial cells and phagocytes, as well as an increased release of cytokines, may eventually enhance the invasive properties of *S. suis*. Further ex vivo experiments as well as in vivo studies should be carried out to reach more definitive conclusions. Finally, this study addressed the possible interactions of these pathogens in the context of respiratory infections. As both *M. hyorhinis* and *S. suis* are able to cause polyserositis and have already been isolated together [16], further studies of the interaction of these pathogens in the context of systemic infections should also be addressed. 

## Figures and Tables

**Table 2:** List of primers and probes for qPCR of mycoplasmas [20].

Gene Name	Forward 5′-3′	Reverse 5′-3′	Probes 5′-3′
*p102* (*M. hyopneumoniae*) *p37* (*M. hyorhinis*)	TAAGGGTCAAAGTCAAAGTC TTCTATTTTCATCTATATTTTCGC	AAATTAAAAGCTGTTCAAATGC TCATTGACCTTGACTAACTG	CY5 *-AACCAGTTTCCACTTCATCGCC-BHQ2 § TXR †-CATCCTCTTGCTTGACTACTCCTG-BHQ2 §

* CY5, cyanine 5, fluorescence reporter dye; † TXR, texas-red, fluorescence reporter dye; § BHQ2, Black Hole Quencher.

**Table 3:** List of primers for RT-qPCR.

Gene Name	Forward 5′-3′	Reverse 5′-3′
*β2M* *PPiA* *IL-6* *CXCL8*	CGTGGCCTTGGTCCTGCTCG TGCAGACAAAGTTCCAAAGACAG ACTCCCTCTCCACAAGCGCCTT TGTGAGGCTGCAGTTCTGGCAAG	TCCGTTTTCCGCTGGGTGGC GCCACCAGTGCCATTATGG TGGCATCTTCTTCCAGGCGTCCC GGGTGGAAAGGTGTGGAATGCGT

## Data Availability

Not applicable.

## Supplementary Materials

The following supporting information can be downloaded at https://www.mdpi.com/article/10.3390/pathogens12070866/s1, Table S1: Titration of M. hyopneumoniae and M. hyorhinis expressed in color-changing units per milliliter (CCU/mL) before and after pre-infection Newborn Pig Tracheal cell line (NPTr), primary pulmonary alveolar macrophages (PAMs) and primary bone-marrow-derived porcine dendritic cells (BM-DCs), Figure S1: Effect of a pre-infection of mycoplasmas on the cytotoxic effect of S. suis on BM-DCs.

## References

[1] Gottschalk M., Segura M., Zimmerman J.J., Karriker L.A., Ramirez A., Schwartz K.J., Stevenson G.W., Zhang J. (2019). Streptococcocis. Diseases of Swine.

[2] Segura M. (2020). *Streptococcus suis* Research: Progress and Challenges. Pathogens.

[3] Gottschalk M., Xu J., Calzas C., Segura M. (2010). *Streptococcus suis*: A new emerging or an old neglected zoonotic pathogen?. Future Microbiol..

[4] Segura M., Fittipaldi N., Calzas C., Gottschalk M. (2017). Critical *Streptococcus suis* Virulence Factors: Are They All Really Critical?. Trends Microbiol..

[5] Obradovic M.R., Segura M., Segales J., Gottschalk M. (2021). Review of the speculative role of co-infections in *Streptococcus suis*-associated diseases in pigs. Vet. Res..

[6] Meng F., Tong J., Votsch D., Peng J.Y., Cai X., Willenborg M., Herrler G., Wu N.H., Valentin-Weigand P. (2019). Viral Coinfection Replaces Effects of Suilysin on *Streptococcus suis* Adherence to and Invasion of Respiratory Epithelial Cells Grown under Air-Liquid Interface Conditions. Infect. Immun..

[7] Saade G., Deblanc C., Bougon J., Marois-Crehan C., Fablet C., Auray G., Belloc C., Leblanc-Maridor M., Gagnon C.A., Zhu J. (2020). Coinfections and their molecular consequences in the porcine respiratory tract. Vet. Res..

[8] Maes D., Sibila M., Kuhnert P., Segales J., Haesebrouck F., Pieters M. (2018). Update on *Mycoplasma hyopneumoniae* infections in pigs: Knowledge gaps for improved disease control. Transbound. Emerg. Dis..

[9] Sibila M., Pieters M., Molitor T., Maes D., Haesebrouck F., Segales J. (2009). Current perspectives on the diagnosis and epidemiology of *Mycoplasma hyopneumoniae* infection. Vet. J..

[10] Wallgren P., Norregard E., Molander B., Persson M., Ehlorsson C.J. (2016). Serological patterns of *Actinobacillus pleuropneumoniae, Mycoplasma hyopneumoniae, Pasteurella multocida* and *Streptococcus suis* in pig herds affected by pleuritis. Acta Vet. Scand..

[11] Kobisch M., Friis N.F. (1996). Swine mycoplasmoses. Rev. Sci. Tech..

[12] Pieters M., Maes D., Zimmerman J.J., Karriker L.A., Ramirez A., Schwartz K.J., Stevenson G.W., Zhang J. (2019). Mycoplasmosis. Diseases of Swine.

[13] Fourour S., Tocqueville V., Paboeuf F., Lediguerher G., Morin N., Kempf I., Marois-Crehan C. (2019). Pathogenicity study of *Mycoplasma hyorhinis* and *M. flocculare* in specific-pathogen-free pigs pre-infected with *M. hyopneumoniae*. Vet. Microbiol..

[14] Buttenschon J., Friis N.F., Aalbaek B., Jensen T.K., Iburg T., Mousing J. (1997). Microbiology and pathology of fibrinous pericarditis in Danish slaughter pigs. Zentralbl. Veterinarmed. A.

[15] Nathues H., Kubiak R., Tegeler R., Beilage E.G. (2010). Occurrence of *Mycoplasma hyopneumoniae* infections in suckling and nursery pigs in a region of high pig density. Vet. Rec..

[16] Kang I., Kim D., Han K., Seo H.W., Oh Y., Park C., Lee J., Gottschalk M., Chae C. (2012). Optimized protocol for multiplex nested polymerase chain reaction to detect and differentiate *Haemophilus parasuis*, *Streptococcus suis*, *and Mycoplasma hyorhinis* in formalin-fixed, paraffin-embedded tissues from pigs with polyserositis. Can. J. Vet. Res..

[17] Auray G., Lachance C., Wang Y., Gagnon C.A., Segura M., Gottschalk M. (2016). Transcriptional Analysis of PRRSV-Infected Porcine Dendritic Cell Response to *Streptococcus suis* Infection Reveals Up-Regulation of Inflammatory-Related Genes Expression. PLoS ONE.

[18] Fourour S., Marois-Crehan C., Martelet L., Fablet C., Kempf I., Gottschalk M., Segura M. (2019). Intra-Species and Inter-Species Differences in Cytokine Production by Porcine Antigen-Presenting Cells Stimulated by *Mycoplasma hyopneumoniae*, *M. hyorhinis,* and *M. flocculare*. Pathogens.

[19] Friis N.F. (1975). Some recommendations concerning primary isolation of *Mycoplasma suipneumoniae* and *Mycoplasma flocculare* a survey. Nord. Vet. Med..

[20] Fourour S., Fablet C., Tocqueville V., Dorenlor V., Eono F., Eveno E., Kempf I., Marois-Crehan C. (2018). A new multiplex real-time TaqMan((R)) PCR for quantification of *Mycoplasma hyopneumoniae*, *M. hyorhinis* and *M. flocculare*: Exploratory epidemiological investigations to research mycoplasmal association in enzootic pneumonia-like lesions in slaughtered pigs. J. Appl. Microbiol..

[21] Kellog D.E., Kwok S., Innis M.A., Gelfand D.H., White T.J., Sninsky J.J. (1990). Detection of human immunodeficiency virus. PCR Protocols: A Guide to Methods and Applications.

[22] Slater J.D., Allen A.G., May J.P., Bolitho S., Lindsay H., Maskell D.J. (2003). Mutagenesis of *Streptococcus equi* and *Streptococcus suis* by transposon Tn917. Vet. Microbiol..

[23] Lecours M.P., Gottschalk M., Houde M., Lemire P., Fittipaldi N., Segura M. (2011). Critical role for *Streptococcus suis* cell wall modifications and suilysin in resistance to complement-dependent killing by dendritic cells. J. Infect. Dis..

[24] Mathieu-Denoncourt A., Letendre C., Auger J.P., Segura M., Aragon V., Lacouture S., Gottschalk M. (2018). Limited interactions between *Streptococcus suis* and *Haemophilus parasuis* in in vitro Co-Infection Studies. Pathogens.

[25] Wang Y., Gagnon C.A., Savard C., Music N., Srednik M., Segura M., Lachance C., Bellehumeur C., Gottschalk M. (2013). Capsular sialic acid of *Streptococcus suis* serotype 2 binds to swine influenza virus and enhances bacterial interactions with virus-infected tracheal epithelial cells. Infect. Immun..

[26] Dang Y., Lachance C., Wang Y., Gagnon C.A., Savard C., Segura M., Grenier D., Gottschalk M. (2014). Transcriptional approach to study porcine tracheal epithelial cells individually or dually infected with swine influenza virus and *Streptococcus suis*. BMC Vet. Res..

[27] Martelet L., Lacouture S., Goyette-Desjardins G., Beauchamp G., Surprenant C., Gottschalk M., Segura M. (2017). Porcine dendritic cells as an in vitro model to assess the immunological behaviour of *Streptococcus suis* Subunit vaccine formulations and the polarizing effect of adjuvants. Pathogens.

[28] Lecours M.P., Segura M., Lachance C., Mussa T., Surprenant C., Montoya M., Gottschalk M. (2011). Characterization of porcine dendritic cell response to *Streptococcus suis*. Vet. Res..

[29] Corsaut L., Misener M., Canning P., Beauchamp G., Gottschalk M., Segura M. (2020). Field Study on the immunological response and protective effect of a licensed autogenous vaccine to control *Streptococcus suis* infections in post-weaned piglets. Vaccines.

[30] Liu W., Zhou D., Yuan F., Liu Z., Duan Z., Yang K., Guo R., Li M., Li S., Fang L. (2019). Surface proteins mhp390 (P68) contributes to cilium adherence and mediates inflammation and apoptosis in *Mycoplasma hyopneumoniae*. Microb. Pathog..

[31] Xiong Q., Zhang B., Wang J., Ni B., Ji Y., Wei Y., Xiao S., Feng Z., Liu M., Shao G. (2016). Characterization of the role in adherence of *Mycoplasma hyorhinis* variable lipoproteins containing different repeat unit copy numbers. Vet. Microbiol..

[32] Auger J.P., Payen S., Roy D., Dumesnil A., Segura M., Gottschalk M. (2019). Interactions of *Streptococcus suis* serotype 9 with host cells and role of the capsular polysaccharide: Comparison with serotypes 2 and 14. PLoS ONE.

[33] Shapouri-Moghaddam A., Mohammadian S., Vazini H., Taghadosi M., Esmaeili S.A., Mardani F., Seifi B., Mohammadi A., Afshari J.T., Sahebkar A. (2018). Macrophage plasticity, polarization, and function in health and disease. J. Cell Physiol..

[34] Savina A., Amigorena S. (2007). Phagocytosis and antigen presentation in dendritic cells. Immunol. Rev..

[35] Chabot-Roy G., Willson P., Segura M., Lacouture S., Gottschalk M. (2006). Phagocytosis and killing of *Streptococcus suis* by porcine neutrophils. Microb. Pathog..

[36] Auger J.P., Boa A.C., Segura M., Gottschalk M. (2019). Antigen I/II Participates in the Interactions of *Streptococcus suis* Serotype 9 With Phagocytes and the Development of Systemic Disease. Front. Cell. Infect. Microbiol..

[37] Segura M.A., Cleroux P., Gottschalk M. (1998). *Streptococcus suis* and group B *Streptococcus* differ in their interactions with murine macrophages. FEMS Immunol. Med. Microbiol..

[38] DeBey M.C., Ross R.F. (1994). Ciliostasis and loss of cilia induced by *Mycoplasma hyopneumoniae* in porcine tracheal organ cultures. Infect. Immun..

[39] Thacker E.L. (2001). Immunology of the porcine respiratory disease complex. Vet. Clin. N. Am. Food Anim. Pract..

[40] Fittipaldi N., Segura M., Grenier D., Gottschalk M. (2012). Virulence factors involved in the pathogenesis of the infection caused by the swine pathogen and zoonotic agent Streptococcus suis. Future Microbiol..

[41] Hsu T., Minion F.C. (1998). Identification of the cilium binding epitope of the Mycoplasma hyopneumoniae P97 adhesin. Infect. Immun..

[42] Leal Zimmer F.M.A., Paes J.A., Zaha A., Ferreira H.B. (2020). Pathogenicity & virulence of *Mycoplasma hyopneumoniae*. Virulence.

[43] Pan Q., Xu Q., Liu T., Zhang Y., Xin J. (2022). *Mycoplasma hyopneumoniae* membrane protein Mhp271 interacts with host UPR protein GRP78 to facilitate infection. Mol. Microbiol..

[44] Park C., Jeong J., Kang I., Choi K., Park S.J., Chae C. (2016). Increased fucosyl glycoconjugate by *Mycoplasma hyopneumoniae* enhances adherences of *Pasteurella multocida* type A in the ciliated epithelial cells of the respiratory tract. BMC Vet. Res..

[45] Ackermann M.R., Debey M.C., Debey B.M. (1991). Bronchiolar metaplasia and Ulex europaeus agglutinin I (UEA-I) affinity in *Mycoplasma hyopneumoniae*-infected lungs of six pigs. Vet. Pathol..

[46] Wang J., Li Y., Pan L., Li J., Yu Y., Liu B., Zubair M., Wei Y., Pillay B., Olaniran A.O. (2021). Glyceraldehyde-3-phosphate dehydrogenase (GAPDH) moonlights as an adhesin in *Mycoplasma hyorhinis* adhesion to epithelial cells as well as a plasminogen receptor mediating extracellular matrix degradation. Vet. Res..

[47] Beier L.S., Siqueira F.M., Schrank I.S. (2018). Evaluation of growth and gene expression of *Mycoplasma hyopneumoniae* and Mycoplasma hyorhinis in defined medium. Mol. Biol. Rep..

[48] Viana F., O’Kane C.M., Schroeder G.N. (2022). Precision-cut lung slices: A powerful ex vivo model to investigate respiratory infectious diseases. Mol. Microbiol..

[49] Haas B., Grenier D. (2018). Understanding the virulence of *Streptococcus suis*: A veterinary, medical, and economic challenge. Med. Mal. Infect..

[50] Varol C., Mildner A., Jung S. (2015). Macrophages: Development and tissue specialization. Annu. Rev. Immunol..

[51] Bieber K., Autenrieth S.E. (2020). Dendritic cell development in infection. Mol. Immunol..

[52] Houde M., Gottschalk M., Gagnon F., Van Calsteren M.R., Segura M. (2012). *Streptococcus suis* capsular polysaccharide inhibits phagocytosis through destabilization of lipid microdomains and prevents lactosylceramide-dependent recognition. Infect. Immun..

[53] Charland N., Kobisch M., Martineau-Doize B., Jacques M., Gottschalk M. (1996). Role of capsular sialic acid in virulence and resistance to phagocytosis of *Streptococcus suis* capsular type 2. FEMS Immunol. Med. Microbiol..

[54] Li J., Wang J., Liu Y., Yang J., Guo L., Ren S., Chen Z., Liu Z., Zhang Y., Qiu W. (2019). Porcine reproductive and respiratory syndrome virus NADC30-like strain accelerates *Streptococcus suis* serotype 2 infection in vivo and in vitro. Transbound Emerg. Dis..

[55] Deeney A.S., Maglennon G.A., Chapat L., Crussard S., Jolivet E., Rycroft A.N. (2019). *Mycoplasma hyopneumoniae* evades phagocytic uptake by porcine alveolar macrophages in vitro. Vet. Res..

[56] Caruso J.P., Ross R.F. (1990). Effects of *Mycoplasma hyopneumoniae* and *Actinobacillus* (*Haemophilus*) *pleuropneumoniae* infections on alveolar macrophage functions in swine. Am. J. Vet. Res..

[57] Bin L., Luping D., Bing S., Zhengyu Y., Maojun L., Zhixin F., Yanna W., Haiyan W., Guoqing S., Kongwang H. (2014). Transcription analysis of the porcine alveolar macrophage response to *Mycoplasma hyopneumoniae*. PLoS ONE.

[58] Bercier P., Gottschalk M., Grenier D. (2020). *Streptococcus suis* suilysin compromises the function of a porcine tracheal epithelial barrier model. Microb. Pathog..

[59] Lun S., Perez-Casal J., Connor W., Willson P.J. (2003). Role of suilysin in pathogenesis of *Streptococcus suis* capsular serotype 2. Microb. Pathog..

[60] Tenenbaum T., Asmat T.M., Seitz M., Schroten H., Schwerk C. (2016). Biological activities of suilysin: Role in *Streptococcus suis* pathogenesis. Future Microbiol..

[61] Votsch D., Willenborg M., Baumgartner W., Rohde M., Valentin-Weigand P. (2021). Bordetella bronchiseptica promotes adherence, colonization, and cytotoxicity of *Streptococcus suis* in a porcine precision-cut lung slice model. Virulence.

[62] Meng F., Wu N.H., Nerlich A., Herrler G., Valentin-Weigand P., Seitz M. (2015). Dynamic Virus-Bacterium Interactions in a Porcine Precision-Cut Lung Slice Coinfection Model: Swine Influenza Virus Paves the Way for *Streptococcus suis* Infection in a Two-Step Process. Infect. Immun..

[63] Bai F., Ni B., Liu M., Feng Z., Xiong Q., Xiao S., Shao G. (2013). *Mycoplasma hyopneumoniae*-derived lipid-associated membrane proteins induce apoptosis in porcine alveolar macrophage via increasing nitric oxide production, oxidative stress, and caspase-3 activation. Vet. Immunol. Immunopathol..

[64] Ni B., Bai F.F., Wei Y., Liu M.J., Feng Z.X., Xiong Q.Y., Hua L.Z., Shao G.Q. (2015). Apoptosis induced by lipid-associated membrane proteins from *Mycoplasma hyopneumoniae* in a porcine lung epithelial cell line with the involvement of caspase 3 and the MAPK pathway. Genet. Mol. Res..

[65] Browning G.F., Marenda M.S., Noormohammadi A.H., Markham P.F. (2011). The central role of lipoproteins in the pathogenesis of mycoplasmoses. Vet. Microbiol..

[66] Kostyal D.A., Butler G.H., Beezhold D.H. (1995). *Mycoplasma hyorhinis* molecules that induce tumor necrosis factor alpha secretion by human monocytes. Infect. Immun..

[67] Kornspan J.D., Tsur M., Tarshis M., Rottem S., Brenner T. (2015). *Mycoplasma hyorhinis* induces proinflammatory responses in mice lymphocytes. J. Basic. Microbiol..

[68] Thanawongnuwech R., Thacker B., Halbur P., Thacker E.L. (2004). Increased production of proinflammatory cytokines following infection with porcine reproductive and respiratory syndrome virus and *Mycoplasma hyopneumoniae*. Clin. Diagn. Lab. Immunol..

