# Supplementary material for: Interactions of Mycoplasma hyopneumoniae and/or Mycoplasma hyorhinis with Streptococcus suis Serotype 2 Using In Vitro Co-Infection Models with Swine Cells
Source: Pathogens. 2023 Jun 22;12(7):866. doi: 10.3390/pathogens12070866 (PMC10383509; doi:10.3390/pathogens12070866)
Supplement: Supplementary file 1 [file pathogens-12-00866-s001.zip › pathogens-2440112-supplementary.pdf]

Table S1. Titration of *M. hyopneumoniae* and *M. hyorhinis* expressed in color-changing units per milliliter (CCU/mL) before and after pre-infection. Newborn Pig Tracheal cell line (NPTr), primary pulmonary alveolar macrophages (PAMs) and primary bone marrow-derived porcine dendritic cells (BM-DC).

Cells	Mycoplasma	Before Pre-Infection (CCU/mL)	After Pre-Infection (CCU/mL)
NPTr	*M. hyopneumoniae*	5×10^8	5×10^8
	M. hyorhinis	4.5×10^6	1.95×10^8
PAMs	*M. hyopneumoniae*	1.95×10^7	2.45×10^8
	M. hyorhinis	3.5×10^6	3.5×10^8
BM-DCs	*M. hyopneumoniae*	4.5×10^7	4.5×10^8
	M. hyorhinis	3.5×10^5	3×10^8

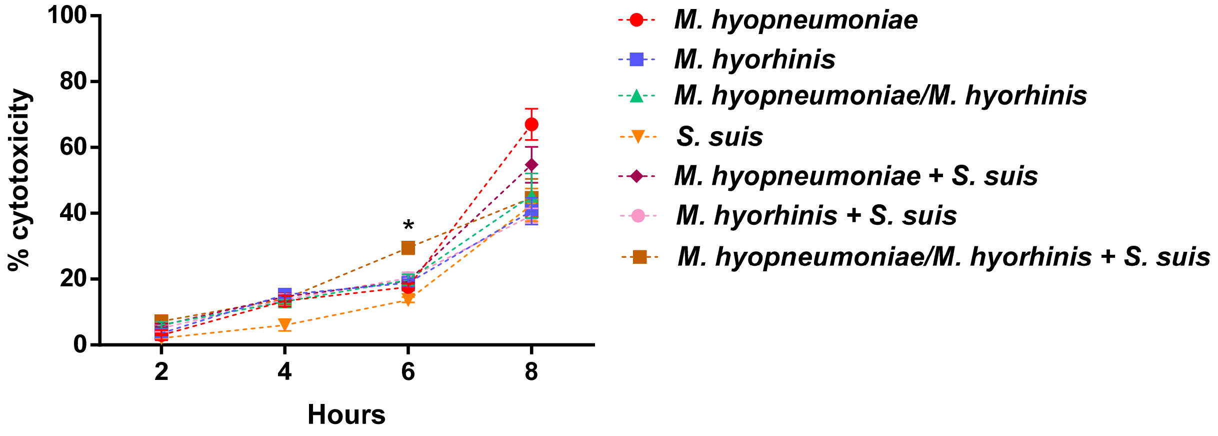

Supplementary Figure S1. Percent cytotoxicity of BM-DCs. After pre-infection of BM-DCs with *M. hyopneumoniae* and/or *M. hyorhinis*, cells were infected with *S. suis* from 2 h to 8 h for BM-DCs at a multiplicity of infection (MOI) of 1. Data represent mean ± SEM (n = 4). * ($p < 0.005$) shows a significant difference between cells pre-infected with mycoplasma + *S. suis* and cells infected with *S. suis* only or cells pre-infected with mycoplasma only using non-parametric Mann-Whitney test.
